# Co-occurrence of Tuberculous Meningitis and Intestinal Perforation in Abdominal Tuberculosis (TB): A Report of a Rare Case From Pakistan

**DOI:** 10.7759/cureus.55132

**Published:** 2024-02-28

**Authors:** Rizwan Ullah, Saad Saeed, Hives Anabel Rafael, Nadia Nishat, Jose Rosario-Curcio, Aiysha Gul

**Affiliations:** 1 Internal Medicine, Hayatabad Medical Complex Peshawar, Peshawar, PAK; 2 Internal Medicine, Nottingham University Hospitals NHS Trust, Nottingham, GBR; 3 Internal Medicine, Universidad Iberoamericana (UNIBE), Santo Domingo, DOM; 4 Family Medicine, Adichunchanagiri Institute of Medical Sciences, Mandya, IND; 5 Medicine, University of Medicine and Health Sciences, Basseterre, KNA; 6 Obstetrics and Gynaecology, Mardan Medical Complex, Mardan, PAK

**Keywords:** report of a rare case, co-occurrence, exploratory laparotomy, intestinal tuberculosis, abdominal tuberculosis, ileal perforation, intestinal perforation, tuberculous meningitis

## Abstract

Tuberculosis (TB) remains a significant global health concern, with millions affected worldwide each year. Extrapulmonary TB, particularly involving the digestive tract and central nervous system, poses distinctive difficulties in both diagnosis and treatment. We report a case involving a 15-year-old girl with a history of intestinal TB on anti-tuberculous therapy who presented with symptoms suggestive of meningitis, along with abdominal pain and distension. Our initial suspicion was tuberculous meningitis, considering the underlining abdominal TB, which was later supported by cerebrospinal fluid analysis showing lymphocytic-predominant pleocytosis and positive acid-fast bacilli staining. Concurrently, the patient developed hemodynamic instability and severe abdominal pain, which on repeat X-rays of the abdomen showed air under the diaphragms, prompting surgical exploration and revealing multiple ileal perforations. Histopathological examination confirmed TB as the cause of perforation. This case highlights the diagnostic and therapeutic complexities of concurrent tuberculous meningitis and intestinal TB perforation. Early recognition and interdisciplinary management are crucial for optimal patient outcomes.

## Introduction

Tuberculosis (TB) continues to pose a significant global health threat, affecting an estimated 10.6 million people worldwide in 2022. This resulted in 1.13 million deaths among human immunodeficiency virus (HIV)-negative people and around 167,000 deaths occurring in individuals co-infected with HIV [1].

Extrapulmonary TB commonly affects various organs, with the digestive tract being one of the primary sites. Approximately one-eighth of all TB cases occur outside the lungs, with abdominal TB constituting 11-16% of these cases. Individuals with HIV have an increased risk, with up to half of HIV-positive individuals developing extrapulmonary TB. Among the most common locations for extrapulmonary TB, the gastrointestinal tract is ranked sixth, following the lymphatic, genitourinary, bone and joint, and meningeal sites. In abdominal TB, the abdomen can be either the primary site of infection or a secondary site due to spread from nearby infected organs, ingestion of sputum, transmission through the bloodstream, consumption of unpasteurized milk, or reactivation of a previously dormant focus [2].

*Mycobacterium tuberculosis* (*M. tuberculosis*) is the primary cause of tuberculous meningitis (TBM) and represents the most common type of TB affecting the brain and spinal cord central nervous system (CNS). TBM can result in neurological complications and mortality if not promptly addressed [3]. Despite its severity, the prevalence of TBM is infrequent in developed nations. Only 100-150 cases are reported yearly in the United States, constituting less than 3% of about 4,100 yearly bacterial meningitis cases [4].

There are different stages of CNS TB. In stage I, individuals are awake, alert, and oriented, displaying no focal neurological deficits. Stage II involves patients with a Glasgow Coma Scale (GCS) score ranging from 14 to 11, accompanied by focal neurological deficits. In stage III, patients have a GCS score of 10 or lower, with or without focal neurological deficits. TBM in adults rarely presents with seizures, and their presence should prompt clinicians to consider other potential diagnoses like bacterial or viral meningitis or cerebral tuberculoma or TBM. However, conversely, TBM commonly presents with seizures in pediatrics, manifesting in up to half of the instances [5].

Diagnosing TBM can be challenging, often based on clinical assessment and initial cerebrospinal fluid (CSF) findings. Specific clinical features such as prolonged symptom duration (more than six days) and CSF leukocytosis, along with the presence of focal neurological deficits, can raise suspicion for TBM. Key CSF features indicative of TBM include lymphocytic-predominant leukocytosis, typically with a total white blood cell (WBC) count ranging from 100 to 500 cells/μL. Protein levels are commonly increased, usually falling within the range of 100-500 mg/dL, while glucose levels tend to be low, often below 45 mg/dL, with a CSF-to-plasma ratio of <0.5 [6].

We reported a case of intestinal TB diagnosed two months prior, with the patient already receiving anti-tuberculous therapy, who presented to an emergency department with symptoms including headache, nausea, vomiting, and positive signs indicative of meningitis. Additionally, the patient complained of abdominal pain and distension. Subsequent evaluation revealed a diagnosis of TBM along with a small bowel perforation attributable to intestinal TB.

## Case presentation

A 15-year-old female patient from Hangu, Pakistan, was previously diagnosed with intestinal TB one and a half months ago and initiated on an anti-tuberculous treatment based on her weight of 45 kg (BMI 19): rifampicin 360 mg once a day (OD), isoniazid 180 mg OD, ethambutol 675 mg OD, and pyrazinamide 900 mg OD. Despite having no other immunocompromised state like HIV, she presented to an emergency department of a tertiary care hospital with a myriad of symptoms, including headache, drowsiness, confusion, fever, nausea, vomiting, abdominal pain, and distension. Upon arrival, her vital signs were stable, but an abdominal examination revealed a rigid abdomen with tenderness throughout. The neurological assessment demonstrated positive signs of neck stiffness and Kernig's sign, while other examination findings were normal.

**Figure 1:**
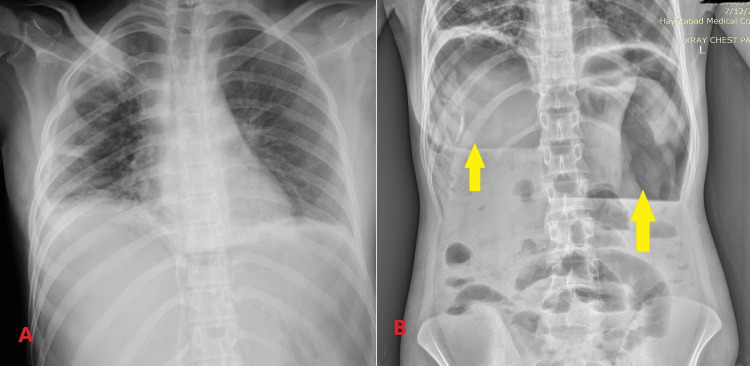
(A) X-ray on arrival shows no abnormality. (B) X-ray of the abdomen on the second day of admission shows a huge collection of air under the bilateral diaphragm (shown by yellow arrows)

During this period, we conducted basic laboratory investigations, all of which returned within normal ranges except for an elevated leukocyte count and an erythrocyte sedimentation rate (ESR) of 65. Abdominal ultrasound revealed aperistaltic gut loops with no other significant findings. Subsequently, an abdominal X-ray was performed, which showed no evidence of intestinal perforations like air under the diaphragm (Figure 1A). Following the exclusion of papilledema, we proceeded with a CSF routine examination, revealing a white blood cell (WBC) count of 567/mm^3^ with predominantly lymphocytes (80%) and protein and glucose levels of 75 mg/dL and 37 mg/dL, respectively. Given that our patient was already receiving anti-tuberculous treatment, we initiated dexamethasone ranging from 0.3 to 0.4 mg/kg/day for a duration of four to six weeks. Subsequently, following this initial phase, we recommended a gradual tapering of the dosage over the course of four to six weeks, reducing it by 2 mg every three to four days until complete discontinuation. On the second day of admission, the patient experienced worsening hemodynamic instability and severe abdominal pain. A repeat abdominal X-ray revealed a significant accumulation of air bilaterally under the diaphragm (Figure 1B).

**Figure 2:**
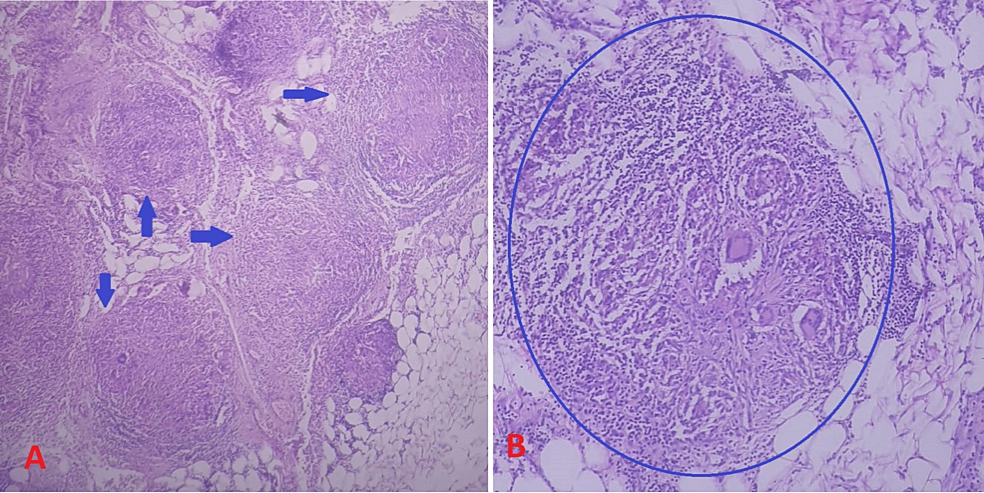
Histopathology shows necrotizing granulomatous inflammations with granuloma formations. (A) shows multiple granulomas (shown by blue arrows). (B) shows single large granulomas (shown by blue encircle)

The patient was urgently referred to the surgical team for an exploratory laparotomy. Upon exploration, the surgical team identified four perforations in the ileum, located at distances of 12 inches, 18 inches, 30 inches, and 72 inches from the ileocecal junction. Following the laparotomy, biopsies were taken for histopathological analysis, which revealed necrotizing granulomatous inflammation and formation of granulomas (Figure 2).

Following the analysis of CSF, imaging results, laparotomy findings, and histopathological examination, the patient was diagnosed with TBM and intestinal perforation resulting from intestinal TB.

For the post-surgical intervention, the patient was transferred to our medical unit for optimal treatment. After 10 days of therapy with rifampicin 360 mg OD, isoniazid 180 mg OD, ethambutol 675 mg OD, and pyrazinamide 900 mg OD along with steroids, the patient exhibited notable improvement, displaying orientation and the absence of active complaints. Subsequently, the patient was discharged with instructions for follow-up appointments with both the medical and surgical teams.

## Discussion

Tuberculous enterocolitis predominantly affects young adults, typically in their third decade of life, with the most commonly affected bowel segments being the ileocecal region, ileum, and colon. Complications of abdominal TB encompass intestinal obstruction, fistula formation, perforation, and bleeding [7]. Research conducted by Talwar et al. identified that 19% of non-traumatic small intestinal perforations among 308 cases were attributed to abdominal TB [7]. Similarly, Badaoui et al. in Switzerland documented 11 cases of intestinal TB perforation; among the 11 cases, 10 occurred in immigrants from TB-endemic countries [8].

Perforation in abdominal TB is typically observed in the terminal ileum and can even manifest in patients undergoing anti-tuberculous therapy [9]. Unfortunately, there are no specific diagnostic tests available for this condition. Plain X-rays have demonstrated free air in only 25-50% of cases. Additionally, approximately 50% of cases with extrapulmonary TB exhibit normal chest radiography. The presence of peritonitis, particularly in patients with chest radiography suggestive of TB, should raise suspicion for a perforated TB ulcer. In cases where patients with abdominal TB present with generalized peritonitis, urgent exploratory laparotomy is needed [10]. In our case, the patient, who was already receiving anti-tuberculous therapy, presented with clinical manifestations suggestive of peritonitis. Despite initial normal findings on chest radiography, the patient's condition deteriorated on the second day, prompting a repeat abdominal X-ray, which revealed a significant accumulation of air under the diaphragm bilaterally. Consequently, we referred the patient to surgery for an exploratory laparotomy.

In 90% of cases, perforation occurs as a solitary event, whereas multiple perforations are seen in 10-40% of cases, which are often linked with a very poor prognosis. Therefore, emergent surgical exploration is imperative. The preferred treatment approach involves resection of the involved segment of the small intestine, followed by end-to-end anastomosis, which has been shown to yield the best outcomes [11]. Simple and direct repair of the small bowel perforation is not recommended because of the increased risk of leakage and fistula creation. Studies have reported a high number of mortality and morbidity rates, exceeding 29.3%; however, this rate can be decreased in patients who undergo surgery within 36 hours of perforation [12].

In our case, the patient presented with four perforations in various segments of the ileum. After 28 hours, the patient underwent an exploratory laparotomy, which resulted in a favorable outcome.

TBM, primarily caused by *Mycobacterium tuberculosis*, is a predominant form of TB affecting the CNS (brain and spinal cord). If TBM is not treated promptly, it can result in significant neurological complications and high mortality [3].

There are different stages of CNS TB. In stage I, individuals are awake, alert, and oriented, displaying no focal neurological deficits. Stage II involves patients with a GCS score ranging from 14 to 11, accompanied by focal neurological deficits. In stage III, patients have a GCS score of 10 or lower, with or without focal neurological deficits. TBM rarely present with seizures in adults and, if they do, should prompt consideration of alternative diagnoses. In contrast, they are frequently observed in pediatric cases, occurring in up to 50% of cases [5]. Our patient exhibited symptoms such as headache, nausea, vomiting, confusion, and positive signs of meningitis (neck stiffness and Kernig's sign).

Diagnosing TBM can pose challenges, often necessitating clinical assessment and initial CSF analysis in the absence of definitive microbiological evidence. Some clinical features, such as prolonged symptom duration (more than six days) and CSF leukocytosis, along with the presence of focal neurological deficits, can raise suspicion for TBM. Key CSF characteristics suggestive of TBM include lymphocytic-predominant leukocytosis, typically with a total WBC count ranging from 100 to 500 cells/μL. Elevated protein levels, usually falling within the range of 100-500 mg/dL, and low glucose levels, often below 45 mg/dL, with a CSF-to-plasma ratio of <0.5 are commonly observed as well [6]. In our case, the routine examination of CSF showed a WBC count of 567/mm^3^, predominantly comprising lymphocytes (80%), with protein levels measuring 75 mg/dL and glucose levels measuring 37 mg/dL. Additionally, acid-fast bacilli (AFB) staining was positive. Magnetic resonance imaging (MRI) is the preferred imaging modality for detecting abnormalities related to TBM, surpassing computed tomography (CT) in its ability to assess the brainstem and spinal cord. T2-weighted MRI is particularly effective in revealing brainstem pathology, while diffusion-weighted imaging (DWI) excels in detecting acute cerebral infarctions caused by TBM [13]. We didn't perform any further imaging study in this emergency case, and after surgery, the patient responded well to treatment.

Our case, presenting with a co-occurrence of both abdominal TB with multiple ileal perforations and TBM, represents a unique scenario not previously documented in medical literature. We pursued surgical intervention for the ileal perforations, while the TBM was managed by the medical team.

## Conclusions

This case underscores the importance of considering extrapulmonary TB, such as TBM and intestinal TB with or without perforation, in patients who present with compatible signs and symptoms, even in those already undergoing anti-tuberculous therapy. Clinicians should maintain a high level of suspicion for extrapulmonary TB and its complications, especially in endemic regions or in patients with risk factors such as HIV infection or recent immigration from TB-endemic areas. Prompt diagnosis and management are crucial, as delayed recognition can lead to severe complications and poorer outcomes. Additionally, our case highlights the need for a multidisciplinary approach involving both medical and surgical teams for the comprehensive management of complex cases involving multiple organ systems affected by TB. Finally, this case emphasizes the importance of vigilance and thorough evaluation in patients with TB to ensure timely intervention and optimize patient outcomes.

## References

[1] (2023). Tuberculosis. https://www.who.int/news-room/fact-sheets/detail/tuberculosis.

[2] Sharma SK, Mohan A (2004). Extrapulmonary tuberculosis. Indian J Med Res.

[3] Verdon R, Chevret S, Laissy JP, Wolff M (1996). Tuberculous meningitis in adults: review of 48 cases. Clin Infect Dis.

[4] Thigpen MC, Whitney CG, Messonnier NE (2011). Bacterial meningitis in the United States, 1998-2007. N Engl J Med.

[5] Farinha NJ, Razali KA, Holzel H, Morgan G, Novelli VM (2000). Tuberculosis of the central nervous system in children: a 20-year survey. J Infect.

[6] Kumar R, Singh SN, Kohli N (1999). A diagnostic rule for tuberculous meningitis. Arch Dis Child.

[7] Talwar S, Talwar R, Prasad P (1999). Tuberculous perforations of the small intestine. Int J Clin Pract.

[8] Badaoui E, Berney T, Kaiser L, Mentha G, Morel P (2000). Surgical presentation of abdominal tuberculosis: a protean disease. Hepatogastroenterology.

[9] Seabra J, Coelho H, Barros H, Alves JO, Gonçalves V, Rocha-Marques A (1993). Acute tuberculous perforation of the small bowel during antituberculosis therapy. J Clin Gastroenterol.

[10] Makanjuola D, al Orainy I, al Rashid R, Murshid K (1998). Radiological evaluation of complications of intestinal tuberculosis. Eur J Radiol.

[11] Gilinsky NH, Voigt MD, Bass DH, Marks IN (1986). Tuberculous perforation of the bowel. A report of 8 cases. S Afr Med J.

[12] Veeragandham RS, Lynch FP, Canty TG, Collins DL, Danker WM (1996). Abdominal tuberculosis in children: review of 26 cases. J Pediatr Surg.

[13] Pienaar M, Andronikou S, van Toorn R (2009). MRI to demonstrate diagnostic features and complications of TBM not seen with CT. Childs Nerv Syst.

